# Association between polymorphic markers human leucocyte antigen-G and tumour necrosis factor alpha and susceptibility to recurrent miscarriages among Bulgarian women

**DOI:** 10.4274/tjod.galenos.2020.48107

**Published:** 2020-04-06

**Authors:** Mariya Levkova, Trifon Chervenkov, Mari Hachmeriyan, Lyudmila Angelova

**Affiliations:** 1 Medical University Varna, Department of Medical Genetics, Varna, Bulgaria; 2 St. Marina Hospital, Laboratory of Medical Genetics, Varna, Bulgaria; 3 St. Marina Hospital, Laboratory of Clinical Immunology, Varna, Bulgaria

**Keywords:** HLA-G, insertion, deletion, TNF-alpha, GA variant, recurrent miscarriage

## Abstract

**Objective:**

To analyze the role of 14 base pair (bp) insertion (ins)/deletion (del) and tumour necrosis factor alpha (TNF-α) G/A polymorphisms as risk factors for spontaneous miscarriage in patients with two or more unsuccessful pregnancies and a group of control women with at least two normal live births.

**Materials and Methods:**

To investigate the role of these mutations, 50 patients with two or more idiopathic recurrent miscarriages and 50 normal fertile women were tested for the presence of human leucocyte antigen-G (HLA-G) 14 bp ins/del and TNF-α -308 G/A variants. The frequencies of the studied polymorphisms were compared between the two groups.

**Results:**

Individuals with a history of miscarriages had a significantly higher prevalence of 14 bp insertion alleles compared with control patients (p=0.04). There was also a two times higher relative risk for miscarriage among carriers of this variant. No statistical difference in allele frequencies of the TNF-α -308 G/A polymorphism was established between controls and study patients (p=0.78).

**Conclusion:**

The 14 bp ins HLA-G variant could be associated with a higher risk for unsuccessful pregnancy according to the results from the study. There is no association between the studied TNF-α -308 GA polymorphism and rate of spontaneous abortions.

**PRECIS:** We have investigated the role of HLA-G and TNF Alpha variants as risk factors for recurrent miscarriages among Bulgarian women with fertility issues.

## Introduction

Recurrent miscarriage is described as three or more consecutive pregnancy losses^(1)^. It is estimated that unsuccessful pregnancies make up around 30% of all conceptions^(2)^. There are various factors for miscarriage such as anatomic, endocrine, immunologic, and genetic. Nevertheless, the reasons often remain unknown^(3)^.

The fetus could be defined as a semi-allograft due to the presence of paternal human leucocyte antigen (HLA) class I molecules in the trophoblast, which invade the decidua basalis^(4)^. In order to prevent a potential miscarriage, the maternal immune system must accept the fetus and immunologic tolerance should be established^(5)^. HLA-G and tumour necrosis factor-alpha (TNF-α) molecules are both involved in this immunomodulation by regulating the functions of natural killer (NK) cells and the cytotoxic T-lymphocytes^(6,7)^.

The expression of HLA-G was first reported in the fetal trophoblast cells of the placenta^(8)^ and it has immunosuppressive functions; it inhibits proliferation and impairs the functions of T cells and NK cells, and induces the apoptosis of activated CD8+ T cells^(g)^. Also, HLA-G binds to the killer cell immunoglobulin-like receptor 2Ll4 (KIR2DL4) found on the surface of the decidual NK cells, and assists the remodeling of spiral arteries. This allows enough blood flow to the fetus and its normal development^(10)^.

However, there are several different polymorphisms found in the HLA-G locus that could have an impact on these functions. One of them is the insertion/deletion (ins/del) of a 14 base pair (bp) polymorphism in exon 8^(11)^. Although the ins leads to a more stable RNA transcript, in individuals who are homozygous for the 14 bp ins polymorphism, there are decreased levels of soluble HLA-G^(12)^. In people who have the del, there are higher levels of this molecule in the plasma, which could explain the possible role of the insertion polymorphism as a risk factor for recurrent miscarriage^(13,14)^.

TNF-α is a proinflammatory cytokine, whose levels might be increased in people carrying the AA polymorphism at position -308 in the TNF-α promotor region compared with carriers of the normal GG variant.^(15)^ This cytokine is considered a key factor for thrombotic events at the maternal uteroplacental blood vessel barrier because of activation of vascular endothelial cell procoagulant in animal models^(16)^. The TNF-α -308 AA polymorphism causes an increased production of the TNF-α cytokine in blood, it might influence the blood flow to the fetus in carriers, and could be associated with pregnancy loss^(17)^.

The aim of the present study was to analyze the role of 14 bp ins/del and TNF-α G/A polymorphisms as risk factors for spontaneous miscarriage in patients with two or more unsuccessful pregnancies, and a group of control women with at least two normal live births.

## Materials and Methods

### Participants

A total of 100 patients were divided into two groups and analyzed between October 2018 and July 2019 in the Laboratory of Medical Genetics in Varna, Bulgaria. Informed consent was obtained from all participants in the study prior to the analysis. The first group consisted of 50 patients with 2 or more idiopathic recurrent miscarriages during the first trimester. The second group included 50 normal fertile women without a previous history of miscarriage. All of the case subjects were without anatomic, microbial, viral, or endocrine diseases, which could explain the miscarriages.

The study was approved by the Ethics Committee of University Hospital St Marina, Varna, Bulgaria Laboratory of Medical Genetics on March 31^st^, 2016 (approval number: 78, date: 25.10.2018). Informed consent was obtained from all participants.

### Methods

**Figure 1:**
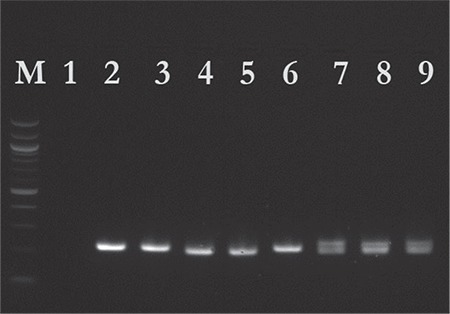
Gel electrophoresis for the detection of the 14 base pair insertion/deletion polymorphism. Lane 1: No template control, lanes 2,3,6: Insertion, lanes 4,5: Deletion, lane 7-9: heterozygote, M: 1 kb DNA ladder

Genomic DNA samples from the individuals were extracted from peripheral blood and genotyped using polymerase chain reaction (PCR) and gel electrophoresis. They were tested for the presence of HLA-G 14 bp ins/del and TNF-α -308 G/A polymorphisms. The HLA-G region was amplified by using a forward primer 5’GTGATGGGCTGTTTAAAGTGTCACC-3’ and a reverse primer 5’- GGAAGGAATGCAGTTCAGCATGA-3’. PCR was performed in a 20 µL reaction, containing 5x HOT FIREPol EvaGreen qPCR Supermix (Solis BioDyne, Estonia), primer mix, and the patient’s DNA. The cycling conditions used were as follows: initial denaturation at 95 °C for 12 minutes, 35 cycles of 94 °C for 30 seconds, 63 °C for 40 seconds, 72 °C for 2 minutes, followed by extension at 72 °C for 7 minutes. All PCR products were evaluated using gel electrophoresis on 2% agarose gels containing ethidium bromide and then visualized under ultraviolet light (Figure 1).

The TNF-α polymorphism was amplified using touch-down real-time PCR using forward 5’TAGGTTTTGAGGGGCAAGG3’ and reverse 5’TAGGTTTTGAGGGGCAAGA3’ primers. PCR was performed in 20 µL reactions, containing 5x HOT FIREPol EvaGreen qPCR Supermix (Solis BioDyne, Estonia), primer mix, and the patient’s DNA. The cycling conditions were initial denaturation at 95 °C for 10 minutes, 5 cycles of 94 °C for 20 seconds, 67 °C for 30 seconds, 72 °C for 45 seconds, 35 cycles of 94 °C for 20 seconds, 63 °C for 30 seconds, 72 °C for 45 seconds, followed by extension at 72 °C for 7 minutes.

### Statistical Analysis

Differences between the two groups were analyzed using the Statistical Package for the Social Sciences software version 23 (IBM, USA) using odds ratio (OR), chi-square, Fisher’s and Kruskal-Wallis tests. ORs were calculated with a confidence interval of 95%. A difference was considered significant at a p< 0.05.

## Results

**Figure 2:**
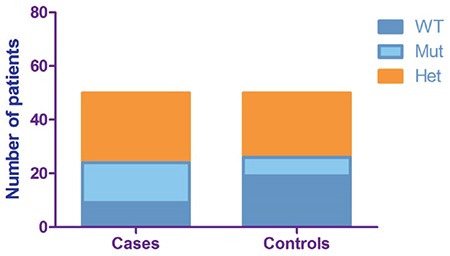
Prevalence distribution of the genotypes of the participants for the 14 base pair insertion/deletion variant in the *HLA-G* gene. WT: Wild type, homozygotes for the deletion allele, Mut: Homozygotes for the insertion allele, Het: Heterozygotes for the insertion/deletion allele

The median age of the women with recurrent miscarriages was 35.0 years (25% percentile - 31.75; 75% percentile - 39.00). All were nulliparous, the mean miscarriage rate was 2.70 [standard deviation =1.147]. The median age in the control group was 33.00 years (25% percentile - 30.75; 75% percentile - 36.00). Nine women (18.0%) from the case group were homozygotes for the wild type (del/del), 15 women (30.0%) were homozygotes for the mutant type (ins/ins), and 26 participants (52.0%) were heterozygotes (del/ins). The frequencies for the control group were 19 (38.0%), seven (14.0%), and 24 (48.0%) women, respectively (Figure 2).

The investigated polymorphism of the HLA-G gene showed a statistically significant difference (p=0.04) compared with the control group (chi-square, degrees of freedom (df): 6.56). The OR was 4.52 and carriers of the ins polymorphism had a 2.12 times higher relative risk for a miscarriage compared with those with the del.

The women were also divided into two other subgroups, depending on their number of miscarriages – two or less and more than two, together with the corresponding HLA-G genotype in order to estimate the role of this polymorphism as an additional risk factor. In the first group, there were two homozygotes for the del, 11 homozygotes for the ins, and 15 heterozygotes. In the second group, there were seven homozygotes for the del, four homozygotes for the ins, and 11 heterozygotes. After applying the chi-square test, there was no statistical difference between the groups (chi-square, df 6.03, p=0.05).

**Figure 3:**
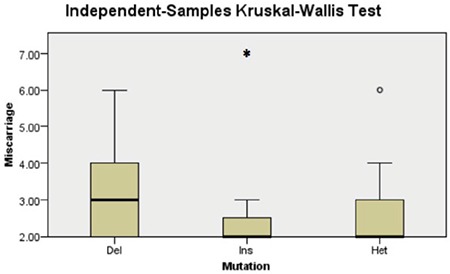
Kruskal-Wallis test for the comparison of the distribution of miscarriage and the different categories of mutation among the participants with recurrent miscarriages. Del: Homozygous for the deletion allele, Ins: Homozygotes for the insertion allele, Het: Heterozygote carriers; *: the highest number of miscarriages for the group of homozygotes for the insertion allele; °: the highest number of miscarriages for the group of heterozygotes

The Kruskal-Wallis test was used to check if the HLA-G ins was associated with multiple miscarriages by comparing the number of spontaneous abortions and the genotypes of patients with recurrent miscarriages. The lowest number of unsuccessful pregnancies was two and the highest was seven. After applying this test, the p value was 0.13 and the null hypothesis was retained (Figure 3).

There were no significant differences in allele frequencies of TNF-α -308 G/A polymorphism between controls and study patients (chi-square, df: 0.08; p=0.78); eight (16.0%) AG heterozygotes and 42 (84.0%) GG homozygotes from the study group, and seven (14.0%) AG heterozygotes and 43 (86.0%) GG homozygotes from the control group. No homozygotes for the mutant allele AA were found in either group.

Fisher’s test was performed in order to evaluate the cumulative effect of having a mutated allele for both polymorphisms. The number of patients from the RSA group and control group who were homozygous for the 14 bp ins and heterozygote GA carriers was two and one, respectively. There were four women who were heterozygotes for both variants from the first group, and five from the control group. No statistically significant difference was found between the two groups (p=0.999).

## Discussion

HLA-G molecules modulate the immune system by inhibiting the activity of cytotoxic T lymphocytes and NK cells, causing apoptosis of activated CD8+ T and CD8+ NK cells, as well as by inhibiting the proliferation of allogenic CD4+ T cells^(18)^. HLA-G could also inhibit the transcription processes in the NK cells, thus protecting the extravillous trophoblast^(18)^.

All these functions of HLA-G illustrate its crucial role in the modulation of the maternal immune response, ensuring tolerance towards the semi-allogenic fetus in order to avoid miscarriage^(19)^.

However, the 14 bp ins variant in the HLA-G gene will lead to alternative splicing and to the lack of 92 bps from the 3’untraslated region^(20)^. Despite the fact that this ins increases the stability of the RNA transcript^(12)^ in individuals who are homozygous for the 14 bp ins, there are lower concentrations of soluble HLA-G in serum compared with people who are homozygous for the 14 bp del^(14,21)^. This might interfere with maintaining pregnancy because low serum concentrations of HLA-G are considered a prognostic marker for increased risk of miscarriage and poor possibility of successful implantation of the embryo after in vitro fertilization^(19,22)^.

According to our results, the 14 bp ins variant is more common among women with recurrent miscarriages. Moreover, women who have this polymorphism have an approximately two times higher relative risk for a miscarriage, compared with controls. These findings correlate with another study, which concluded that the group of women with recurrent miscarriages showed a higher frequency of the ins allele in HLA-G, both in single and double copies^(23)^. Another research group found that the total number of ins alleles was higher among participants with fertility issues, but the number of heterozygotes was the highest^(24)^.

We also applied the Kruskal-Wallis test in order to estimate if carrying the HLA-G ins allele would increase the number of miscarriages, but according to the results, this variant had no impact on the amount of sponaneous abortions. In addition, after comparing the role of the HLA-G 14 bp ins variant as a risk factor for more than two miscarriages, there was no statistical difference. One limitation of the study is that the sample size was small. If more people were included, a statistical difference might have been established because the p value was close to the level of significance for both tests. However, the 14 bp ins polymorphism could be considered as a risk factor for a pregnancy loss itself, regardless of the number of miscarriages.

Even though the mechanisms for this are not clear, in a study conduncted in Denmark, there was a correlation between the 14 bp ins/del polymorphism and fetoplacental growth. The authors concluded that mothers who were homozygous for the 14 bp del gave birth to babies with higher birthweight compared with the children of mothers homozygous for the 14 bp ins^(25)^. However, the exact mechanisms of the protective effect of the HLA-G del remain to be determined.

The TNF-α -308 GA variant is also considered as a risk factor for miscarriages. In vitro experiments showed that this substitution resulted in higher activity of the transcription and increased levels of TNF-α in lipopolysaccharide-stimulated whole blood cell cultures^(26)^. People who are carriers of the TNF-α -308 GA genotype also have a higher plasma concentration of TNF-α compared with those with the GG genotype^(27)^. However, during pregnancy there is normally an increased production of Th2 or immunosuppressor cytokines such as interleukin 4 (IL-4), IL-10, and the levels of the proinflammatory cytokines IL-2 and TNF-α are decreased^(28)^. Also, because TNF-α could activate NK cells in animal models^(29)^ and blood clotting by increasing the expression of prothrombinase fibrinogen-like protein 2 fgl2,^(30)^ it was assumed that the TNF-α -308 GA variant could be a risk factor for recurrent abortions.

Nevertheless, we found no statistical difference betweeen the two studied groups for the TNF-α -308 GA polymorphism. The distrubiton of the mutated alleles was similar between the two groups. This is in agreement with the results from another study among 132 women with recurrent miscarriages, which concluded that TNF-α -238, but not the TNF-α -308 GA variant could have a potential impact^(31)^. The role of the studied polymorphism in TNF-α was excluded by another research group, which stated that there were higher levels of TNF-α during early pregnancy in women with recurrent miscarriages, but this was due to other variants in the TNF-α gene and not to the TNF-α -308 GA polymorphism^(32)^.

Moreover, according to our results, mutations in both locuses of the HLA-G and TNF-α gene did not differ between the two groups and no cumulative effect was observed.

### Study Limitations

Further studies are needed to define other factors that increase the risk of recurrent miscarriages. Confirmation of the data on a larger sample size could provide a better insight into the possible protective effect of del/del homozygotes for the HLA-G polymorphism.

## Conclusion

Recurrent miscarriages may be due to various etiologic factors, but the 14 bp ins HLA-G variant could be associated with a higher risk for unsuccessful pregnancy according to the results from our study. There is no association between the studied TNF-α -308 GA polymorphism and the rate of spontaneous abortions among Bulgarian women. However, the 14 bp ins variant could be included in the test panel for women with recurrent miscarriages.
